# Patrones electrocardiográficos de alto riesgo en pacientes con síndrome coronario agudo

**DOI:** 10.47487/apcyccv.v1i4.82

**Published:** 2020-12-31

**Authors:** Diego Alejandro Echeverri-Marín, Cristhian F. Ramírez-Ramos, Andrés Felipe Miranda-Arboleda, Gustavo Adolfo Castilla-Agudelo, Clara Inés Saldarriaga-Giraldo

**Affiliations:** 1 Departamento de Cardiología Clínica, Clínica CardioVID. Medellín, Colombia. Departamento de Cardiología Clínica Clínica CardioVID Medellín Colombia; 2 Departamento de Cardiología Clínica, Clínica CardioVID y Universidad Pontificia Bolivariana. Medellín, Colombia. Universidad Pontificia Bolivariana Departamento de Cardiología Clínica Clínica CardioVID Universidad Pontificia Bolivariana Medellín Colombia; 3 Departamento de Medicina Interna, Universidad Pontificia Bolivariana. Medellín, Colombia. Universidad Pontificia Bolivariana Departamento de Medicina Interna Universidad Pontificia Bolivariana Medellín Colombia; 4 Departamento de Cardiología Clínica y Falla Cardiaca, Clínica CardioVID y Universidad Pontificia Bolivariana. Universidad de Antioquia. Medellín, Colombia. Universidad Pontificia Bolivariana Departamento de Cardiología Clínica y Falla Cardiaca Clínica CardioVID Universidad Pontificia Bolivariana Medellín Colombia

**Keywords:** Infarto del Miocardio, Electrocardiografía, Mortalidad, Myocardial Infarction, Electrocardiography, Mortality

## Abstract

El infarto agudo de miocardio es la principal causa de muerte en el mundo, y el electrocardiograma sigue siendo la herramienta diagnóstica para determinar un infarto agudo de miocardio con elevación del segmento ST. A pesar de ello, solo la mitad de los pacientes presenta hallazgos clásicos en el electrocardiograma, compatibles con los criterios de infarto con elevación del ST. Existe un espectro de hallazgos electrocardiográficos que pueden reflejar un fenómeno de oclusión coronaria aguda, el cual debe ser prontamente reconocido por el clínico para ofrecer una terapia de reperfusión temprana.

## Abstract (en)

Acute myocardial infarction is the leading cause of death in the world and the electrocardiogram remains the diagnostic tool for determining an acute myocardial infarction with ST-segment elevation. In spite of this, only half of the patients present classic electrocardiogram findings compatible with the ST-elevation infarction criteria. There is a spectrum of electrocardiographic findings that may reflect a phenomenon of acute coronary occlusion, which should be promptly recognized by the clinician to offer early reperfusion therapy.

La interpretación correcta de los hallazgos electrocardiográficos ante un síndrome coronario agudo es un reto diagnóstico para todo clínico y cobra especial importancia en el ámbito de urgencias, donde el pronto diagnóstico de una oclusión coronaria aguda, y una terapia de reperfusión temprana, disminuye la morbimortalidad de pacientes con infarto agudo de miocardio con elevación del segmento ST (IAMCEST)^1^.

Clásicamente la elevación del segmento ST en el electrocardiograma ha sido reconocido como la interpretación eléctrica de un fenómeno agudo de oclusión de una arteria coronaria epicárdica ^2^. El IAMCEST es definido, ante un contexto clínico apropiado, como la elevación del segmento ST (medido a nivel del punto J) en al menos dos derivadas contiguas: > 2,5 mm en hombres < 40 años; 2 mm en hombres ≥40 años, o 1,5 mm en mujeres en las derivadas V2 y V3, y/o 1 mm en las demás derivadas; siempre y cuando haya ausencia de bloqueo de rama izquierda del haz de His o hipertrofia ventricular izquierda ^3^. A pesar de esto, al menos la mitad de los pacientes con un infarto agudo de miocardio no presentan cambios electrocardiográficos ^4^.

A su vez, se ha reconocido una serie de patrones electrocardiográficos que se refieren como equivalentes a un IAMCEST o infarto agudo de miocardio con hallazgos eléctricos de alto riesgo, que son causados por la oclusión de una arteria epicárdica con un importante territorio miocárdico irrigado ^5^.

A continuación, se describen esta serie de patrones electrocardiográficos de alto riesgo, con el fin de brindarle al clínico una herramienta adicional para identificación de estos y alertarlo de su significado clínico. 

## Oclusión de la primera diagonal

**Figura 1:**
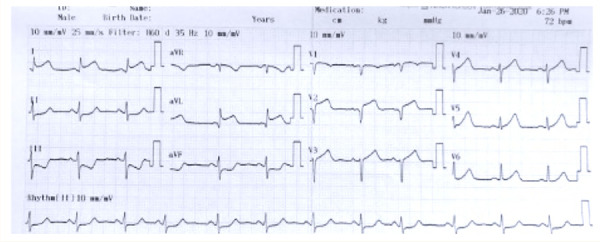
Electrocardiograma de doce derivaciones. Se muestran las anormalidades típicas de cambios del segmento ST en aVL y V2. Además, hay un infradesnivel del segmento ST en DIII y aVF, como supradesnivel del ST en DI.

La oclusión de la primera diagonal, rama de la arteria descendente anterior, se presenta electrocardiográficamente como una elevación significativa del segmento ST en aVL y V2 **(**Figura 1**)** (ver valores previamente descritos, para la elevación del segmento ST); se puede acompañar de T hiperagudas en estas dos derivadas y, ocasionalmente, ondas T negativas en cara inferior ^5^. Esta característica electrocardiográfica se ha denominado como «presentación electrocardiográfica no anatómica», dado que no cumple con los criterios tradicionales de elevación del ST en infarto agudo de miocardio ^5^.

La importancia del diagnóstico precoz de esta entidad radica en que la rama diagonal de la arteria descendente anterior irriga la toda la pared anterolateral del ventrículo izquierdo; la cual es un gran porcentaje de la masa miocárdica del ventrículo izquierdo ^6^^)^ .

## Patrón de De Winter

**Figura 2:**
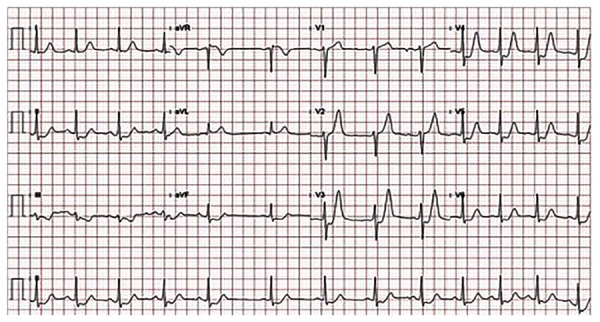
Electrocardiograma de doce derivaciones. Se muestra una depresión con parte terminal ascendente del segmento ST con ondas T positivas y simétricas en V3-V4-V5-V6. Además, hay una depresión horizontal en DII y aVF. Se encuentra una elevación del segmento ST en aVR.

Fue descrito en el 2008 por Robert de Winter y colaboradores ^7^. Este patrón se caracteriza por una depresión ascendente del segmento ST desde el punto J de 1-3 mm en las derivaciones V1-V6 con ondas T positivas simétricas y, eventualmente, una sutil elevación de 1-2 mm del ST en aVR **(**Figura 2**)**. Este raro patrón electrocardiográfico comúnmente se asocia con enfermedad coronaria de un vaso y es estático sin progresión a un patrón de infarto del miocardio con elevación del segmento ST (desaparece solo después de la revascularización). Se ha documentado en el 2% de los infartos de la pared anterior. Un año después de la publicación inicial, el mismo grupo reportó un estudio observacional donde este hallazgo se encontró en el 2% de las oclusiones proximales de la arteria descendente anterior (valor predictivo positivo 100%). Se observó de manera más frecuente en personas jóvenes con dislipidemia ^8^. En Ámsterdam se confirmó igual observación: 1,6% en los infartos anteriores y, de manera invariable, con oclusión proximal o media de la arteria descendente anterior con predominio en hombres ^9^. 

## Patrón de Wellens

**Figura 3:**
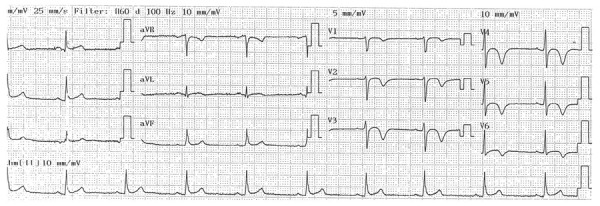
Electrocardiograma de superficie de doce derivaciones. Hallazgos característicos de un patrón de Wellens tipo B con una inversión profunda y simétrica de la onda T en derivaciones V2-V3; hay extensión hasta V4 y V5.

**Figura 4:**
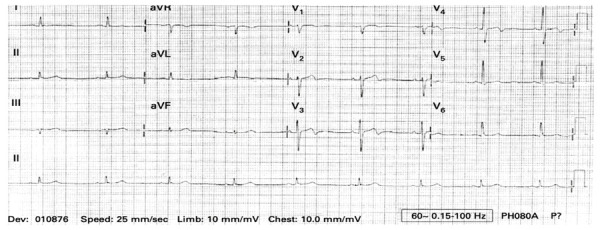
Electrocardiograma de superficie de doce derivaciones. Patrón de Wellens tipo A se evidencia una onda T bifásica con porción terminal negativa en V2-V3-V4.

Este hallazgo electrocardiográfico se caracteriza por una inversión de la onda T en las derivaciones V2-V3 (usualmente en el periodo libre de dolor) en pacientes con historia de síntomas intermitentes del espectro de enfermedad coronaria. Se considera un signo de alarma, ya que representa una lesión proximal crítica de la arteria descendente anterior que se ha relacionado con pobre pronóstico en pacientes a quienes no se les realiza angiografía o revascularización. Los hallazgos se caracterizan por una inversión profunda de la onda T (patrón tipo B; Figura 3) o una onda bifásica positiva-negativa (patrón tipo A; Figura 4), en el cual el segundo componente de la onda T está invertido. El 76% de los pacientes se presenta con el patrón tipo B y el 24% con el patrón tipo A. 

En 1982 De Zwaan *et al.*^10^ reportaron un estudio de 145 pacientes que se presentaron al hospital sin síntomas, pero con angina a menudo *in crescendo*; empeoramiento de la angina o posinfarto, que característicamente tenían una mínima, o sin elevación, de enzimas cardiacas. El 18% (n=26) tuvo una inversión de la onda T en V2-V3 a la admisión o en las primeras 24 h. El valor pronóstico de estos hallazgos se desconocía para 9 de los 26 pacientes; sin embargo, ocho pacientes desarrollaron infarto de miocardio a pesar de que los síntomas se resolvieron con manejo médico. De la población restante, a trece se les realizó angiografía encontrando enfermedad coronaria en doce casos. Todos tenían una estenosis de 90%, o más, de la arteria descendente anterior. En un estudio posterior con 1260 pacientes que se presentaron con angina inestable, 14% tuvo hallazgos similares en la onda T como en el trabajo original. La angiografía de reveló 50%, o más, de estenosis de la arteria descendente anterior en todos los pacientes y lesión proximal a la segunda perforante septal en el 83%. El grado de obstrucción fue de 79-85% y la oclusión total en el 18% ^11^.

## Elevación de ST en aVR e infradesnivel > 6 derivadas

El cuadro clínico de presentación de la mayoría de los pacientes con un síndrome coronario agudo, con este patrón electrocardiográfico, es con choque cardiogénico, edema pulmonar, arritmias que comprometen la vida, y/o muerte súbita ^12^. Esto se debe a que este patrón electrocardiográfico puede ser la representación de la oclusión del tronco principal izquierdo, el cual irriga el 75% de la masa ventricular izquierda ^13^. También puede encontrarse en pacientes con enfermedad coronaria multivaso o con compromiso proximal de la descendente anterior ^14^.

**Figura 5:**
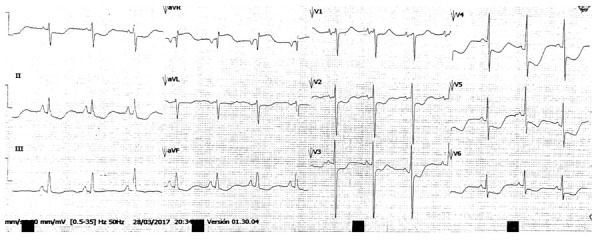
Infradesnivel del ST > 1 mm en todas las derivadas y supradesnivel del ST en aVR > 1 mm.

Los hallazgos electrocardiográficos de este patrón consisten en elevación del ST en aVR e infradesnivel del ST > 1 mm en más de seis derivadas del electrocardiograma de superficie (Figura 5). Ante estos hallazgos no se debe esperar a la toma de biomarcadores de lesión miocárdica para proceder con urgencia a una estratificación invasiva ^3^. 

## Infarto inferobasal

Desde el año 2008 el Dr. Antony Bayés de Luna propuso un cambio dogmático y, gracias a los estudios de resonancia magnética cardiaca, pudo demostrar que el corazón carece de pared posterior, y que lo que se denominaba previamente **«**posterior**»** corresponde al segmento inferobasal, que es la continuación de la pared inferior del ventrículo izquierdo por debajo del surco auriculoventricular, y que descansa sobre el diafragma ^15^. La cuarta definición universal de infarto avala este cambio y reemplaza el término posterior por infarto inferobasal ^16^.

Se cree que en hasta el 20% de los infartos agudos de miocardio (IAM) puede presentarse compromiso inferobasal, principalmente acompañando infartos inferiores y laterales. El compromiso inferobasal verdadero es menos frecuente y solo se presenta en 3,3% de todos los infartos. La mayoría de los pacientes que se presentan con este tipo de infarto tiene compromiso de la arteria circunfleja, seguido por la arteria coronaria derecha ^17^.

**Figura 6:**
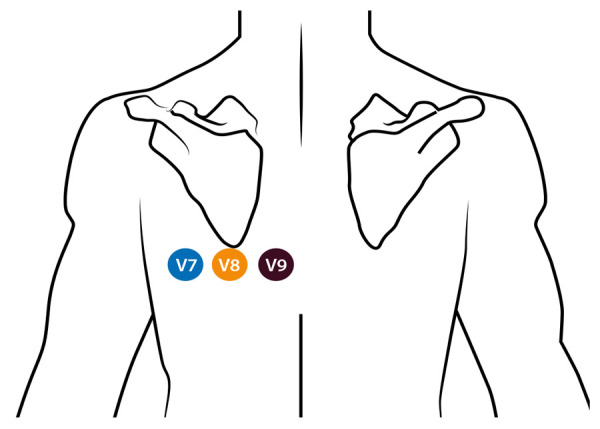
Ubicación de las derivadas posteriores. V7 iría en la en quinto espacio intercostal con la línea axilar posterior, V8 quinto espacio intercostal con línea escapular media a la altura del ángulo de la escapula y V9 en el quinto espacio intercostal con la línea paravertebral izquierda. Para tomar estas derivadas en los electrocardiógrafos convencionales se deben desconectar otras derivadas precordiales p. ej. v4 a v6 y ponerlos en este lugar. Tomado de: https://litfl.com/posterior-myocardial-infarction-ecg-library/

**Figura 7:**
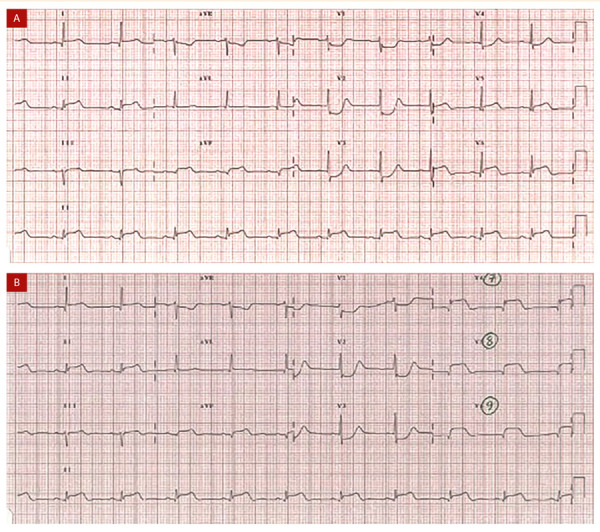
A) Características en ECG de doce derivadas que hacen sospechar compromiso inferobasal: infradesnivel en V1 a V3, R alta de V1 a V3 y R/S que en V2, T positiva en V2 y V3. También se evidencia elevación del ST en DII, DIII y aVF B) supradesnivel del ST de V8 a V9. Tomado de: https://litfl.com/posterior-myocardial-infarction-ecg-library/

El segmento inferobasal no está representado en el electrocardiograma convencional de doce derivada. Para su diagnóstico es necesario utilizar quince derivadas que incluyan derivadas posteriores V7, V8 y V9 **(**Figura 6**)**; cualquier elevación del ST mayor de 0,5 mm en estas será positiva para infarto ST inferobasal. Esto se explica porque en este caso hay una pérdida de las fuerzas eléctricas que van en sentido posterior y, por ende, los cambios típicos de elevación del ST solo se verán en estas derivadas. El punto clave para detectar este tipo de infartos es que cuando en un ECG de doce derivaciones se identifique infradesnivel del ST en V1, V2 o V3, que corresponde a la imagen en espejo, se tomen siempre, y de manera inmediata, derivadas posteriores. También debe evaluarse el QRS donde se aprecie una relación R/S > 1 en V1 y V2 como espejo de una posible Q en curso en V7 a V9. Dependiendo del momento de presentación puede haber ondas T hiperagudas en las derivadas precordiales **(**Figura 7**)**^18^.

## Bloqueo de rama derecha del haz de His

La presencia de bloqueos de rama en el contexto de infarto agudo varía entre 1,6 a 10,9% ^19^, sin un predominio significativo entre la incidencia los bloqueos de rama izquierda o derecha. La presencia de bloqueos de rama es un factor importante en el enfoque de pacientes que se presentan en contexto de infarto, pues este puede interferir con la interpretación de los cambios de repolarización y retrasar las intervenciones terapéuticas.

El bloqueo de rama derecha no afecta la interpretación de las alteraciones de repolarización que habitualmente afectan el ventrículo izquierdo. Sin embargo, su presencia en el escenario de infarto agudo es un factor de mal pronóstico que ha venido cobrando mayor fuerza en los últimos años; los pacientes con IAM y bloqueo de rama derecha del haz de His (BRDHH) tienen dos veces mayor riesgo de muerte a 30 días ^20^.

Los resultados de un metaanálisis reciente demuestran que la presencia de BRDHH de nueva aparición se asocia con infartos extensos, y mayor tendencia a tener complicaciones como choque cardiogénico, arritmias ventriculares y bloqueos AV ^21^. Esto podría explicarse porque la rama derecha del haz de His está irrigada por la descendente anterior o ramas septales proximales de esta; por lo tanto, la presencia de este bloqueo indica oclusión proximal de la descendente anterior.

**Figura 8:**
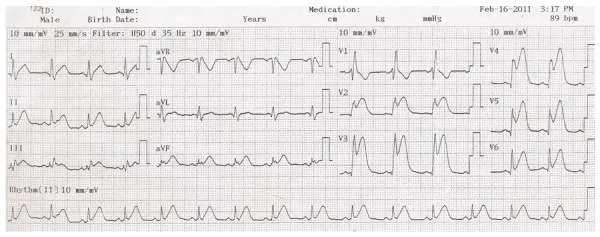
Se observa elevación del segmento ST de V2 a V6 (infarto anterior extenso), además morfología de bloqueo de rama derecha por rSR en V1 y qR en V6.

Las características electrocardiográficas de este patrón incluirán elevación del segmento ST en pared anterior V2 a V6 y morfología del QRS compatible con BRDHH por presencia de rSR en V1 y qRs en V5 o V6 **(**Figura 8**)**.

## Bloqueo de rama izquierda del haz de His

Aproximadamente 7% de los IAM cursan con BRIHH. Diferentes estudios han demostrado que los pacientes con IAM y bloqueo de rama izquierda del haz de His (BRIHH) al momento de la presentación tienen un peor pronóstico por aumento de la mortalidad 2 a 3 veces mayor que quienes no tiene BRIHH. Algunas explicaciones para esto radican en que los pacientes con este patrón electrocardiográfico con frecuencia tienen más años, sufren de falla cardiaca, son más comórbidos y tienen con mayor frecuencia enfermedad multivaso; además, cuando un BRIHH se presenta en contexto de IAM se correlaciona con oclusión de la DA proximal afectando un gran territorio. Otro factor relacionado con peor pronóstico en esta población es que la presencia de BRIHH dificulta la interpretación de las alteraciones de repolarización típicas de IAM generando retraso en el diagnóstico y la implementación de medidas terapéuticas ^22^. No obstante, también se ha notado que en pacientes con dolor torácico y BRIHH, con frecuencia se hace un diagnóstico inadecuado de infarto y, hasta en la mitad de los casos, se han tratado como infarto llegando a recibir, incluso, trombólisis, pero sin tener ninguna evidencia del mismo en análisis retrospectivos ^23^.

Se han propuesto diferentes criterios y puntajes para el diagnóstico de IAM en presencia de BRIHH. Los más frecuentes son:

### Criterios de Sgarbossa

**Figura 9:**
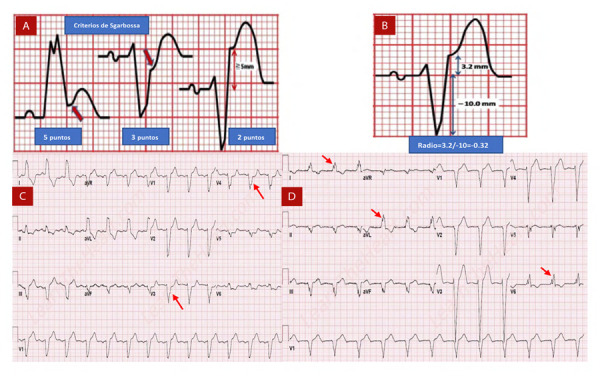
Representación esquemática de diferentes criterios electrocardiográficos para diagnóstico de infarto en presencia de BRIHH. A) Criterios de Sgarbossa; B) Criterio de Smith; C) Criterio de Cabrera - Friedland, las flechas rojas muestran una muesca prominente de >50 ms de duración en la rama ascendente de la onda S de V3 a V4. D) Criterio de Chapman - Pearce, las flechas rojas muestran melladura prominente en la rama ascendente de la onda R en DI, aVL y v6.

En 1996, Sgarbossa *et al.* publicaron los criterios para el diagnóstico de infarto en presencia de BRIHH basados en la información recogida en un estudio clínico controlado denominado el GUSTO-I ^24^. De esta publicación se propusieron como criterios diagnósticos **(**Figura 9a**)**:

Modificado de: https://journalfeed.org/article-a-day/2019/enhancing-sgarbossa-criteria-for-stemi y https://www.healio.com/cardiology/ learn-the-heart/ecg-review/ecg-topic-reviews-and-criteria/left-bundle-branch-block-chapmans-sign

1. Elevación del segmento ST ≥1 mm y concordante con el QRS (5 puntos).
2. Depresión del segmento ST ≥1 mm en V1, V2 o V3 (3 puntos).
3. Elevación del segmento ST ≥5 mm y discordante con el complejo QRS (2 puntos).

El diagnóstico de infarto se hace con la presencia de tres puntos o más, siendo el más poderoso predictor de infarto el criterio de elevación concordante del ST con una especificidad del 98%, pero una sensibilidad del 20%, es decir, que son de gran utilidad cuando están presentes, pero su ausencia no descarta infarto. El criterio de elevación discordante del ST ≥5 mm se encontró como el de menor rendimiento diagnóstico^25^.

### Criterio de Smith o Sgarbossa modificado

En el año 2012, Smith publicó lo que se ha considerado el criterio de Smith o Sgarbossa modificado, este se hizo con el fin de mejorar el rendimiento diagnóstico del último criterio de Sgarbossa de la elevación discordante del ST ≥5 mm, e introducía el concepto de la proporcionalidad de la elevación del ST en relación con el tamaño del QRS. El criterio Smith propone una relación entre la elevación del segmento ST dividido el tamaño de la S, ambas medidas desde el punto J. Se considera positivo si este resultado es ≤ -0.25 **(**Figura 9b**)**. En contexto de infarto la presencia de este criterio se asoció en el 58% de los casos con oclusión de la descendente anterior proximal vs. 8% en quienes no lo cumplieron. Este cambio incrementó la sensibilidad a 91% y la especificidad a 90%, comparado previamente con 41 y 85%, respectivamente ^26^.

### Criterio de Cabrera - Friedland

Muesca prominente de >50 ms de duración en la rama ascendente de la onda S de V3 a V4 **(Figura 9c)**.

### Signo de Chapman-Pearce

Melladura prominente en la rama ascendente de la onda R en DI, aVL o v6 **(**Figura 9 d**)**.

**Figura 10:**
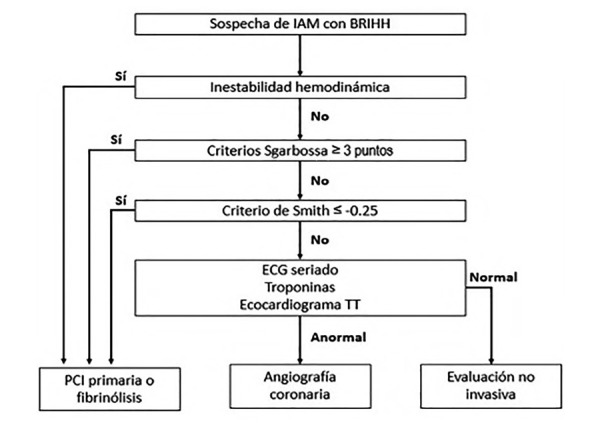
Algoritmo diagnóstico en pacientes con BRIHH y sospecha de IAM.

**Figura 11:**
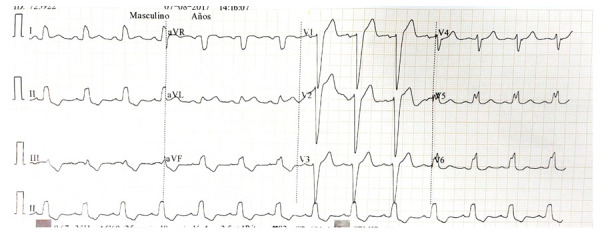
ECG de un paciente con BRIHH y criterio de Sgarbossa positivo por elevación concordante del ST en DI, aVL (5 puntos), además se insinúa signo de Chapman-Pearce en V6.

En la Figura 10 se resume un algoritmo diagnóstico para pacientes con bloqueo de rama izquierda y sospecha de infarto, el patrón electrocardiográfico del paciente con IAM y BRIHH se ejemplifica en la Figura 11**.**

## Conclusión

Conocer el espectro de hallazgos electrocardiográficos compatibles con una oclusión coronaria aguda en el contexto de un evento coronario agudo, es fundamental para el diagnóstico temprano y pronta reperfusión coronaria, con lo que conlleva a la disminución de complicaciones y mortalidad de los pacientes con estos hallazgos. 
